# Effects of Propylene Carbonate on the Properties of Epoxy Resin/Carbon Fiber Multilayer Material Used for Underwater Artifact Extraction

**DOI:** 10.3390/polym17212891

**Published:** 2025-10-29

**Authors:** Hao Wang, Qijun Huang, Xiangna Han

**Affiliations:** 1 National Center for Archaeology, Beijing 100013, China; haow21@126.com; 2 Key Laboratory of Archaeomaterials and Conservation, Ministry of Education, Institute for Cultural Heritage and History of Science and Technology, University of Science and Technology Beijing, Beijing 100083, China; huangqj1117@126.com

**Keywords:** fragile artifacts, epoxy resin, multilayer material, temporary consolidation

## Abstract

The use of epoxy resin/carbon fiber multilayer materials can effectively prevent damage to artifacts during underwater archaeological artifact extraction. However, the high viscosity of epoxy resin limits the coating ability and curing quality. To solve this problem, propylene carbonate (PC) was used as a diluent to reduce the viscosity and improve the properties. This study investigated the gel time, curing time, curing degree, and mechanical strength of the cured products by varying PC concentrations. It was found that the PC significantly enhanced the flowability of epoxy resin blends, extending the gel time from 22 min to 38 min, with minimal impact on the curing time. The glass transition temperature (Tg) ranged from 25.80 °C to 12.28 °C. Compared to E44/PC0, PC reduced the CTE values in the weft direction. The multilayer materials exhibited good temperature stability. The cured product had good mechanical properties and toughness, and could wrap irregular artifacts. The E44/PC10 formulation was used to prepare the epoxy resin/carbon fiber multilayer material. Waterlogged wooden artifacts were successfully extracted from 13 m deep calm water, using the multilayer material.

## 1. Introduction

Marine archaeology is an important method to study ancient cultural heritage [1]. A large number of ancient relics are preserved in the ocean, including shipwrecks [2], submerged cities [3], and various artifacts [4]. Underwater artifacts are precious parts of humanity’s shared history and heritage as they provide vital evidence and information about the interaction of humans with oceans, lakes and rivers [5]. Although in situ preservation is the primary strategy for protecting underwater artifacts, it is sometimes necessary to recover fragile artifacts under controlled and safe conditions to prevent irreversible damage and to ensure their long-term conservation in a more stable environment [6].

However, marine environments present significant challenges for artifact extraction [7]. First, during long-term submersion, artifacts are subjected to various factors such as marine salt infiltration [8,9], human activities [10], biological erosion [11,12], and damage caused by marine sediments [13]. Some underwater artifacts are extremely fragile, and unable to be extracted directly. Secondly, scattered artifacts such as ropes and ornaments, which have a certain arrangement, contain significant archaeological information. Direct extraction may cause the loss of archaeological information. Additionally, divers can only operate for 30 to 40 min underwater and dive twice a day. Finally, the murky, low-visibility conditions of the marine environment further complicate and endanger underwater operations.

Currently, the main methods used for underwater artifact extraction are direct extraction and large-scale caisson techniques. The direct extraction method, which involves placing artifacts into boxes or sealed bags, is straightforward and suitable for small, durable artifacts. The large-scale caisson technology, on the other hand, is employed for extracting large underwater artifacts. The “Nanhai I” shipwreck was salvaged using this method [14,15]. However, these methods cannot be applied for fragile underwater artifacts. To ensure the safety and integrity of artifacts during extraction and transportation, consolidation materials are typically used to temporarily consolidate the artifacts and their remains into a solid unit. This method not only protects the artifacts, but also preserves their original information. Once the artifacts are safely extracted from the water, the temporary consolidation materials are removed, and further protection is provided under controlled laboratory conditions. Therefore, it is important to study materials that can consolidate fragile artifacts underwater and are easy to use. The European SASMAP project (Development of tools and techniques to Survey, Assess, Stabilise, Monitor And Preserve underwater archaeological sites) utilizes materials such as epoxy composites, 3M bandages, carbomer gels, and liquid nitrogen for the extraction of underwater artifacts. These materials can cross-link to form a rigid layer in the underwater environment, which can protect fragile artifacts [16,17]. The Heritage Conservation Laboratory of Zhejiang University has developed a PU sponge modified with superhydrophobic multi-walled carbon nanotubes, exhibiting strong hydrophobicity. The sponge is combined with sublimation materials such as veratraldehyde and 4-dihydrochromanone, to extract archaeological wooden artifacts and bamboo strips under laboratory conditions [18,19,20,21,22,23]. These studies are effective attempts at underwater artifact extraction to solve the problem such as safety of extraction and the preservation of archaeological information. However, most of the newly developed technologies still rely on ideal laboratory conditions or calm underwater environments, and their operational efficiency often decreases significantly under real marine conditions characterized by turbidity, high water pressure, and irregular artifact morphology.

Among these technologies, epoxy resin stands out, due to its rapid curing rate, ease of preparation, and strong environmental adaptability, making it a promising candidate for applications in underwater archaeology. Nevertheless, in practical use, epoxy resins also present several challenges, such as complex handling procedures, intense exothermic reactions during curing, and poor conformity to irregular artifact surfaces, which limit their direct application in underwater artifact consolidation and extraction. Therefore, it is necessary to modify epoxy resin and develop a portable, artifact-friendly, and highly conformable temporary solidification material and extraction technique to meet the demands of safe retrieval and long-term preservation of cultural relics under complex marine environments.

To address these issues, a high-toughness epoxy resin/carbon fiber multilayer material was prepared. The multilayer material consists of four layers: epoxy blend, carbon fiber cloth, and two layers of plastic film. The main components of the epoxy blend include epoxy resin E44, underwater amine hardener JH-5553, and accelerator DMP-30. The rigid protective layer formed upon curing can safely encase artifacts and surrounding sediments for integrated extraction. The multilayer was prepared on land and immediately used underwater, greatly reducing the time of operations. Additionally, the formulation can be flexibly adjusted according to environmental conditions, ensuring that the cured material maintains high toughness and realizes the random wrapping of irregular artifacts. Currently, this material has been successfully simulated to extract artifacts in the laboratory and has been applied at the underwater archaeological site of Shengbeiyu Island [24]. Although epoxy resin E44 offers good processing performance, it has a high viscosity, ranging from 20,000 to 40,000 mPa·s. Such high viscosity tends to cause uneven mixing and coating difficulties of the epoxy resin blend, which affects the performance and stability of the multilayer material [25].

The addition of diluents is a common method for reducing the viscosity of epoxy resins. Diluents are mainly classified into two categories: non-activated diluent and active diluent. Compared to non-reactive diluents, reactive diluents are mostly low-molecular-weight epoxy resins. They not only reduce viscosity, but can also participate in reactions, allowing the flexible chains from diluent to enter the cross-linked network and thereby influence its properties [26,27]. PC is an excellent reactive diluent that can rapidly decrease the viscosity of epoxy resins. Additionally, it can act as a dispersant for fillers, enhancing the uniformity of epoxy blends. When used in water and humid environments, PC exhibits good water resistance and chemical stability, which can improve the stability and durability of epoxy resins underwater [28]. To clarify the role of PC in the reaction and impact on the products, this study investigates the effect of PC amount on the curing degree, thermal properties, and mechanical properties of epoxy resin blends. Finally, the multilayer materials incorporated into PC are employed for the extraction of archaeological wooden artifacts from a calm water depth of 13 m.

## 2. Materials and Methods

### 2.1. Materials

Bisphenol A type epoxy resin (E44) was obtained from Baling Petrochemical Co., Ltd. (Yueyang, Hunan, China). The epoxy content varies between 213 and 244 equiv/g. E44 has a viscosity of about 20,000–40,000 mPa·s. Propylene carbonate (PC) diluent and Tris (dimethylaminomenthyl) phenol (DMP30) were both supplied by Suzhou Rainbow Stone Composite Materials Co., Ltd. (Suzhou, Jiangsu, China). The hardener (JH-5553), a modified amine hardener with an amine content of 110 equiv/g, was sourced from Jinhong Glue Industry (Yiwu, Zhejiang, China). The waterlogged woods were obtained from the Nanhai NO. I shipwreck (Yangjiang, Guangdong, China). Samples are supplied by the National Center for archaeology.

### 2.2. Preparation of Epoxy Resin/Carbon Fiber Multilayer Material

**Figure 1:**
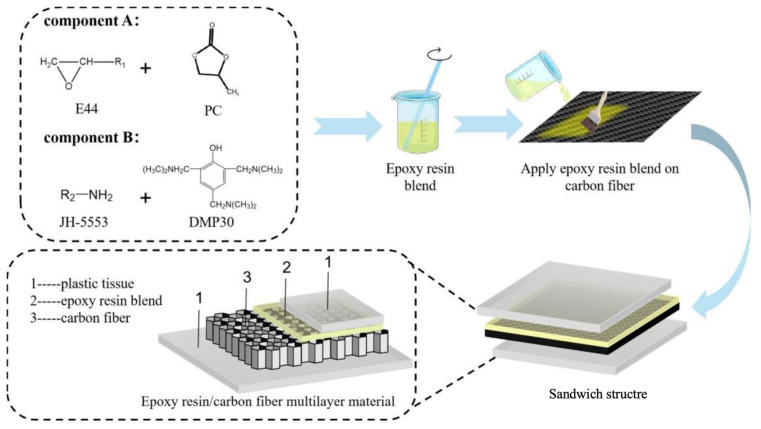
The preparation of epoxy resin/carbon fiber multilayer material.

The solution mixing approach was employed to prepare epoxy resin blends containing 0, 5, 10, 15, and 20 Phr (parts per hundred resin) PC. First, PC was mixed with 100 Phr of epoxy resin while being constantly stirred. Next, 60 Phr of JH-5553 and 3 Phr of DMP30 were added to the E44/PC blends. The amount of component was calculated based on the weight of E44. The formulation of epoxy resin blends is detailed in Table 1. Finally, a carbon fiber sheet was placed between two plastic films and coated with the epoxy resin blend on both sides through resin infiltration. Figure 1 illustrates the preparation of epoxy resin/carbon fiber multilayers.

### 2.3. Characterization and Testing

All specimens tested in this study were pure epoxy resin samples.

#### 2.3.1. Gel Time

To evaluate the gelation behavior of the epoxy resin blends, the viscosity evolution during curing was monitored using an NDJ-5S digital viscometer (Yueping, Shanghai, China). Measurements were conducted at a constant temperature of 20 ± 1 °C using a No. 4 rotor at a rotation speed of 60 rad/min. The viscosity of each blend was recorded every 1 min until the resin reached a non-flowable state. The relaxation time was calculated by fitting the viscosity data of blends using the Maxwell exponential growth equation [29]. The equation is given as follows:

η(t) = η_0_ + A_0_ exp(t/t_n_) (1)

where η(t) is the viscosity, η_0_ is initial viscosity, A_0_ is a parameter and t_n_ is the relaxation time for an increase in viscosity.

#### 2.3.2. Fourier Transform Infrared Spectrometer

To identify chemical structure evolution and monitor functional group changes during the curing process, Fourier transform infrared spectroscopy was performed using a Thermo Fisher Nicolet iS5 spectrometer (Thermo Scientific, Waltham, MA, USA), used to characterize the infrared spectra of the epoxy resin blends during the curing process. The epoxy resin blends were cured underwater, and FT-IR spectra were recorded in the wave number range of 400–4000 cm^−1^ using the ATR mode, with a scanning resolution of 16 cm^−1^.

#### 2.3.3. Thermogravimetric Analysis

To evaluate the thermal stability and decomposition behavior of the cured materials, thermogravimetric analysis was conducted using an SDT-Q600 synchronized thermal analyzer (TA, Delaware, DE, USA). The temperature was programmed to increase from 30 to 800 °C at a rate of 10 °C/min in a nitrogen atmosphere. This temperature range was chosen to cover the full decomposition profile of organic components, avoiding unwanted oxidation effects.

#### 2.3.4. Glass Transition Temperature

To determine the glass transition temperature (Tg) and assess the crosslinking characteristics of the cured epoxy blends, DSC measurements were carried out using a DSC25 differential scanning calorimeter (TA, Delaware, DE, USA), in the temperature range of −40~80 °C, with a heating rate of 20 °C/min in a nitrogen atmosphere. This range was selected to encompass the expected Tg region of epoxy systems while preventing thermal decomposition at elevated temperatures.

#### 2.3.5. Thermal Expansion Performance

To examine and quantify the thermal expansion behavior, the coefficient of linear thermal expansion was measured with a TMA7100 thermomechanical analyzer (HITACHI, Tokyo, Japan). The tests were carried out in the temperature range from -30 °C to 60 °C in a nitrogen atmosphere. This temperature range corresponds to the typical service conditions of polymeric consolidants. The test load was 0.1 N; the heating rate of the samples was 5 °C/min. The samples were tested in the weft and warp directions. Based on the following formula, the coefficient of thermal expansion was calculated:

(2) $$a=\frac{x-x_{0}}{x_{0}∆T}=\frac{∆X}{x_{0}∆T}$$

where X is sample length after temperature change, X_0_ is initial length, a is coefficient of thermal expansion, and ∆T is temperature increase.

#### 2.3.6. Mechanical Testing

The testing specimens were prepared for mechanical testing according to the China Standard Test Method for Mechanical properties of Resin, GB/T 2567-2008 [30]. The tensile specimens had an overall length of 200 mm, a width of 20 mm, a thickness of 4 mm, and a gauge length of 60 mm with a narrow section width of 10 mm. The flexural specimens were 150 mm in length, 15 mm in width, and 4 mm in thickness. The epoxy resin blends were gently poured into the designated specimen mold to avoid the formation of excessive air bubbles and allowed to cure underwater for 48 h. Tensile and flexural strengths were determined using a universal materials testing machine (SANS, Shenzhen, China). Each sample was tested five times, and the average of the five measurements per sample is presented in this report. The tensile tests were conducted at a strain rate of 10 mm/min, while the flexural tests were performed at a strain rate of 10 mm/min with a support span of 60 mm. Each sample was tested five times, and the average of the five measurements per sample is presented in this report.

#### 2.3.7. Scanning Electron Microscope

To investigate the fracture morphology and interfacial characteristics of the cured epoxy blends, SEM imaging was performed using a Regulus 8100 cold-field emission scanning electron microscope (HITACHI, Tokyo, Japan). The tensile-fractured surfaces were embrittled in liquid nitrogen and sputter-coated with a thin gold layer to enhance conductivity.

#### 2.3.8. Degradation of Archaeological Wood

Analyzing the archaeological wood used in simulation experiments can better evaluate the effect of using epoxy resin/carbon fiber multilayer materials. The flexural test of archaeological wood samples was carried out using the TMA7100 thermomechanical analyzer (HITACHI, Tokyo, Japan), together with the quartz flexural probe and three-point flexural fixture. The test was carried out at room temperature with an initial load of 0.1 mN and a loading speed of 5 mN/min until the specimen was broken. The fracture patterns were measured by VHX-6000 ultra-depth three-dimensional microscope (KEYENCE, Osaka, Japan). Scanning electron microscope images of the samples were obtained using a Regulus 8100 cold-field scanning electron microscope after gold spraying.

#### 2.3.9. How to Use Multilayer Material

**Figure 2:**
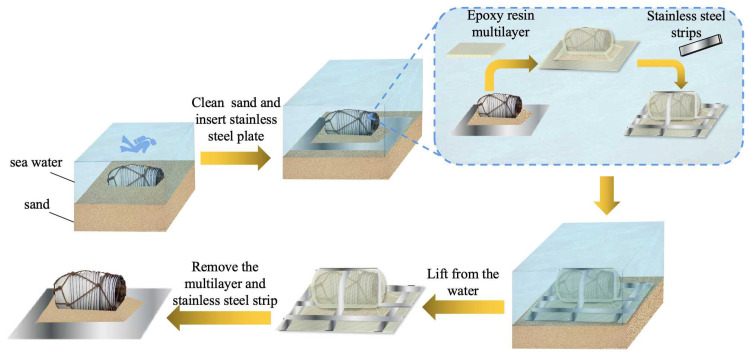
The extraction process of underwater artifacts with epoxy resin/carbon fiber multilayer.

The schematic diagram of the extraction process is shown in Figure 2. A brush was used to clean the sediment around the artifacts. The artifacts were consolidated by inserting a steel plate at their base and enveloping them in the multilayer material. Additionally, steel strips were applied around the edges to provide further reinforcement. After the epoxy resin curing process was completed, the artifacts could be consolidated and extracted. Once the package was removed from the water, the steel strips and multilayer material were taken off.

## 3. Results

### 3.1. Curing Process

**Figure 3:**
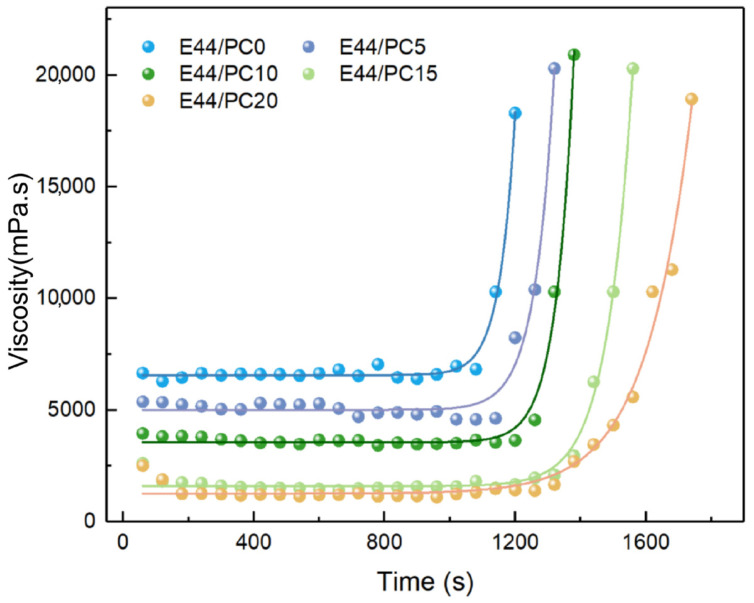
The viscosity of different epoxy resin gels.

Gelation serves as the inception of the curing reaction, and determines the workable time. Epoxy resin undergoes a transformation from the liquid to solid state due to the crosslinking reaction that takes place during the curing process. Initially, the reaction mixture is a liquid with stable viscosity. The viscosity decreases as PC content increases. After gelation, the viscosity increases rapidly because of the network formation. The trend of viscosity is shown in Figure 3. From the figure, it is clear that the viscosities of all blends fit Maxwell’s exponential equation quite well. The relaxation times obtained from the simulation are listed in Table 2. Compared to E44/PC0, the relaxation times increase with the addition of PC, which may be due to PC reducing the active amine concentration in the epoxy resin blend. As a result, the collision frequency between epoxy and amine groups decreases, leading to a lower overall reaction rate and a reduced crosslinking density. The viscosity of epoxy resin blends is closely related to their coating performance. Furthermore, the exothermic reaction during curing accelerates gelation, the process that has been predicted using the William–Landel–Ferry (WLF) equation [31]. A viscosity of 10,000 mPa·s was used as the critical processing limit, at which point the blend loses its flowability and coating capability [32]. The workable times of the epoxy resin blends are shown in Table 2. With the addition of PC, the workable time increases, varying between 22 and 38 min. The PC helps in preparing low-viscosity, homogeneous epoxy resin blends, and enhances the penetration ability of the epoxy resin blend on carbon fibers, facilitating the formation of a more tightly bonded layer. Additionally, the extended gel time also provides ample time for preparing multilayer materials.

**Figure 4:**
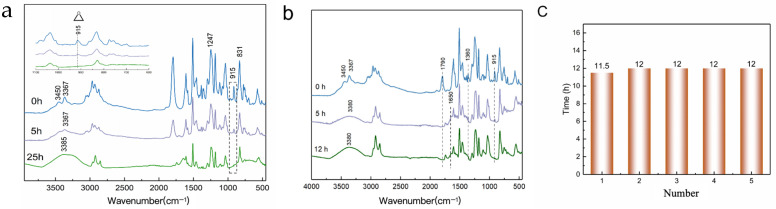
(**a**) FT-IR of E44/PCo epoxy resin-blend curing process; (**b**) FT-IR of E44/PC5 epoxy resin-blend curing process; (**c**) curing time of different epoxy resin blends.

PC, as a reactive diluent participating in the reaction, has an impact on the cured product. To elucidate the role of PC in the epoxy–amine reaction, FT-IR was used to monitor changes in the functional groups within the blends. Figure 4a shows the IR spectrum of the E44/PC0 blend. The E44/PC0 exhibited distinct peaks for primary and secondary amines at 3450 cm^−1^ and 3367 cm^−1^, which originate from the amine hardener. These two peaks disappear after the reaction is complete, while a peak for hydroxyl (OH) groups emerges at 3370 cm^−1^. Additionally, the disappearance of the peak for epoxy groups at 915 cm^−1^ confirms the completion of the epoxy–amine reaction [33]. Figure 4b shows the FTIR spectra of the E44/PC5 blend. The carbonyl group of PC is characterized by a peak at 1793 cm^−1^, and gradually disappears as the reaction progresses; the stretching vibration peak of amide appears at 1650 cm^−1^ [34].

The curing process of the epoxy resin blends can be illustrated by tracking the changes in the characteristic peak of the epoxy group at 915 cm^−1^, as shown in Figure 4c [35]. The addition of PC does not significantly change the curing time of the blends. PC primarily participates in the formation of small-molecule intermediate products at the beginning, which is negligible compared with the time required for the construction of the macromolecular network. The curing time of epoxy resin blends remains stable at 11 to 12 h. As a reactive diluent, PC becomes part of the cross-linked network through chemical participation, but does not affect the curing rate of the epoxy system.

### 3.2. Thermogravimetric Properties

**Figure 5:**
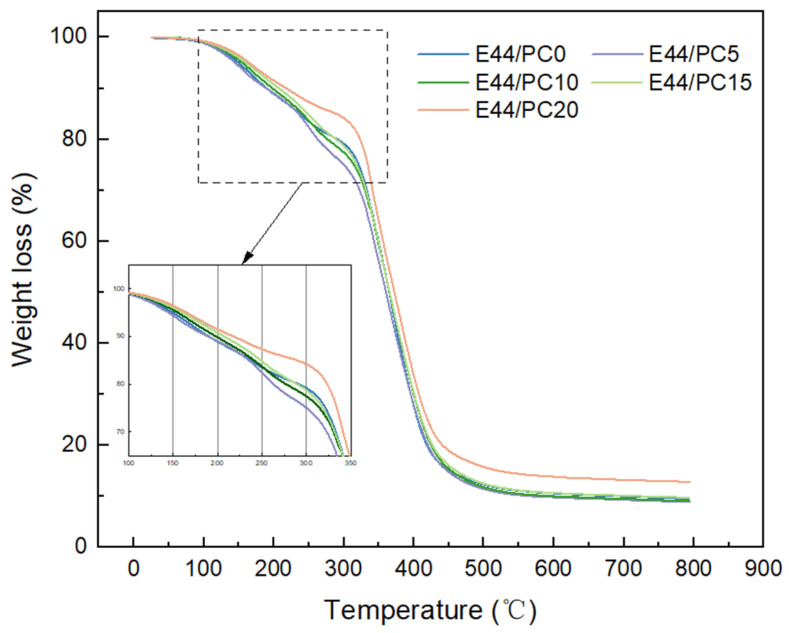
The TG curve of different cured epoxy resin products.

The difference in degradation behavior of the epoxy resin containing various diluent contents was clearly observed from the different thermogravimetric thermograms. Based on the number of stages in the TG thermograms shown as Figure 5, the weight loss processes of the epoxy resins were considered as several stages (Table 3). The PC containing samples shows a weight loss (designated as stage 1 weight loss) at temperatures lower than 180 °C. This stage of weight loss was not observed for E44/PC0, which is attributed to the degradation of end groups formed by the curing reaction with PC upon heating. Furthermore, the unreacted amine groups in the system caused the second-stage weight loss [36]. During this stage, the weight loss also decreases as the PC content increases. The TG thermogram shows a significant weight loss within the range of 280–500 °C. In this stage, the main chain of the cured product breaks down. The weight loss can be used to indicate the curing degree of epoxy resin. Therefore, PC diluent participates in the construction of the epoxy main chain and promotes the curing of epoxy resin. With increasing PC content, the carbon residue of the system gradually decreased. The introduction of PC simultaneously incorporated flexible aliphatic chains into the network, reducing the proportion of aromatic structures and consequently decreasing the amount of carbonized products during pyrolysis [37]. A high curing degree can form stable structures, thereby exhibiting excellent adaptability in the complex environment of underwater archaeology.

**Figure 6:**
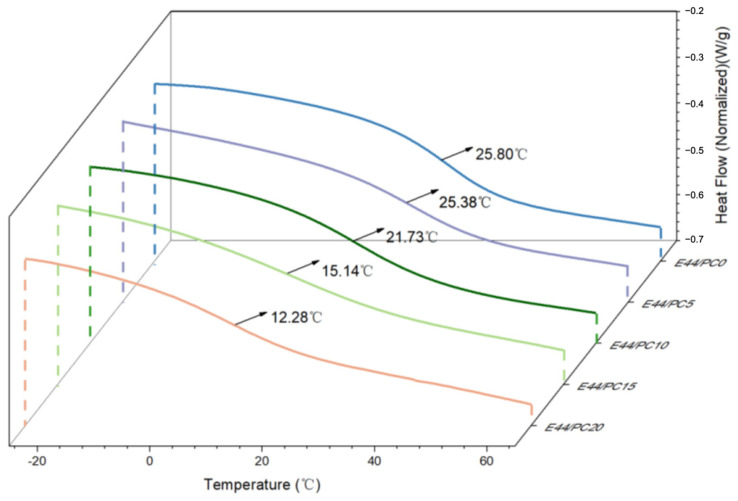
Tg of different cured epoxy resin products.

The glass transition temperature (Tg) of the epoxy resin is determined as a method of monitoring the degree of crosslinking in the system. These data are shown in Figure 6, which illustrates the fact that the Tg of epoxy resin steadily decreases as the content of PC increases, from 25.80 °C for E44/PC0 to 12.28 °C for E44/PC20. The decrease in the Tg is attributed to the participation of the PC in the curing reaction, which promotes the formation of flexible chain segments within the crosslinked network and simultaneously increases the free volume of the system, thereby enhancing the mobility of molecular chains. In addition, the presence of small-molecule diluents weakens the intermolecular interactions between the matrix chains, thereby reducing the crosslinking density. The variation in the Tg of the epoxy resin provides a means to regulate the material properties according to seawater temperature. By adjusting the PC content, epoxy formulations with different Tg values can be prepared, ensuring that the cured resin remains in a highly elastic state at the target marine temperature. This tunable flexibility allows the material to conform closely to irregular and fragile artifact surfaces, enhancing adhesion and reducing interfacial stress, thereby improving its performance during artifact extraction and preservation.

To ensure the stability of the epoxy resin, CTE measurements were conducted to evaluate expansion or contraction behavior under temperature changes. The test results are listed in Table 4. The specimens exhibit significant anisotropic behavior. In the glassy state, the CTE values of all samples are similar, the internal network of the epoxy resin is in a “frozen” state, and PC does not affect thermal stability. As the temperature increases, the specimen transitions from a glassy state to a rubbery state, leading to changes in thermal expansion behavior: the CTE in the warp direction remains unaffected, while it increases in the weft direction [38]. Compared to E44/PC0, the addition of PC effectively reduces the CTE values in the weft direction. The high crosslink density from PC participation limits thermal motion of molecular chains. As the amount of PC increases, the number of flexible chains also rises, intensifying the thermal motion of the molecular chains and consequently leading to an increase in the CTE value. Therefore, an appropriate amount of PC can effectively improve the thermal stability of the epoxy resin, making multilayer materials more suitable for marine environments with large diurnal temperature fluctuations. It is particularly important for epoxy resins with a low glass transition temperature.

### 3.3. Mechanical Properties

Table 5 shows the mechanical properties of a sample, obtained by using the mean values calculated from reproducible results. All specimens exhibit lower tensile strength than that of E44/PC0, with tensile strength decreasing as the PC content increases. This trend aligns with the Tg of epoxy resin. The strain at break for each specimen, as indicated in Table 5, increases significantly with a PC content greater than 15 Phr. The improvement in the diluent can be attributed to the participation of the diluent in the curing reaction, which alters the crosslinked network structure, consistent with the behavior observed for other reactive diluents [39,40]. Table 5 also indicates that the flexural behavior is significantly different from the tensile behavior. An appropriate amount of PC (<10 Phr) improves the flexural strength. This improvement may be due to increased dispersion caused by PC, reducing the stress concentration of the specimen under flexural load. With a PC content of >15 Phr, the flexural strengths show a downward trend, which may be due to excessive PC increasing the flexible segments, affecting the flexural strength.

**Figure 7:**
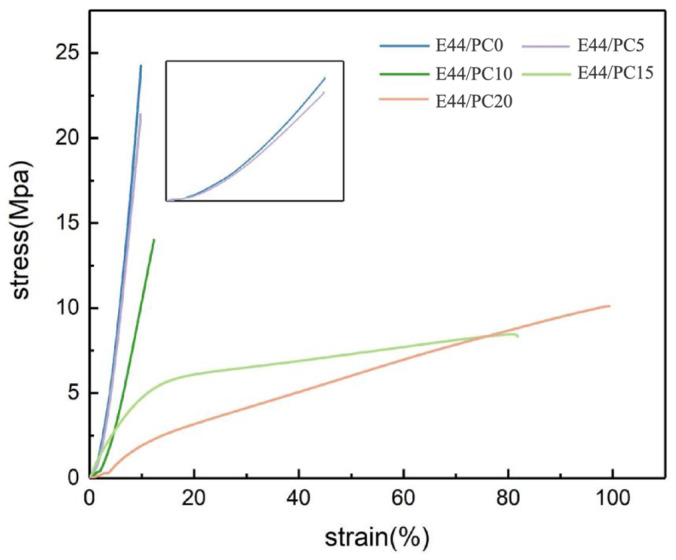
Stress–strain curve of epoxy resin cured products (20 °C).

Figure 7 shows typical engineering stress–strain curves obtained from these tests. It is evident that the fracture mechanisms of the epoxy resin are influenced by the PC content. E44/PC0, E44/PC5, and E44/PC10 exhibit brittle fracture characteristics. The curve slope decreases as PC content increases, continuously, and the samples gradually transition from brittle fracture to ductile fracture [41,42].

**Figure 8:**
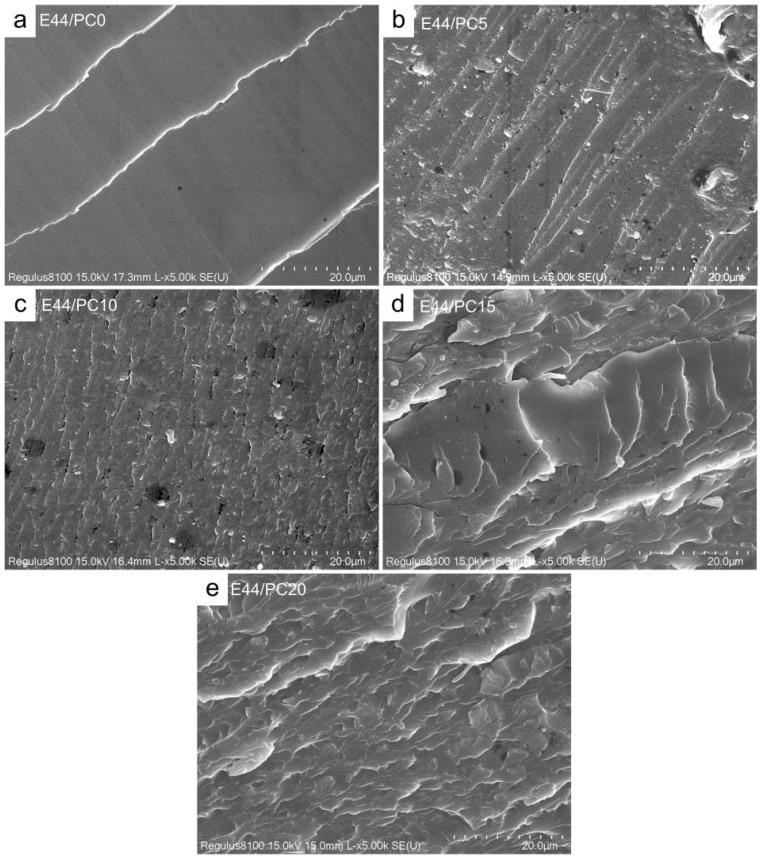
SEM image of fracture surface of cured epoxy resin products (×5000). (**a**) E44/PC0; (**b**) E44/PC5; (**c**) E44/PC10; (**d**) E44/PC15; (**e**) E44/PC20.

The fracture surfaces of epoxy resin samples were analyzed by SEM. As can be seen in Figure 8a, the fracture surface of E44/PC0 is very smooth, except for several river-like lines, indicating that no large-scale plastic deformation had occurred during fracture. Figure 8b shows the fracture surface morphology of E44/PC5. It can be seen that the fracture surface becomes rough, indicating that ductile fracture appears. In Figure 8b–e, the roughness of the fracture surface appears to be increased with the increasing content of PC. PC, as a separate phase closely bonded to the matrix, hinders crack propagation during fracture, leading to a rougher fracture surface [43]. This finding further demonstrates the excellent compatibility of PC with epoxy resin.

### 3.4. Application

**Figure 9:**
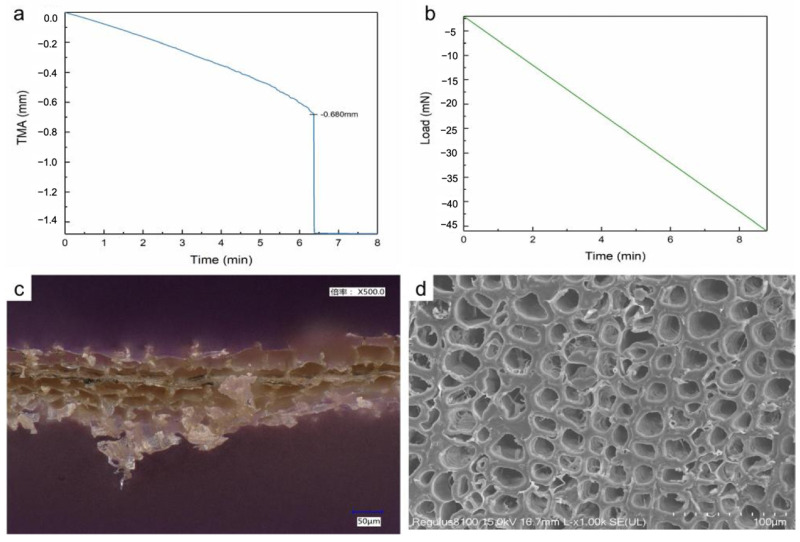
(**a**) Load-time and displacement-time test curves; (**b**) negative values represent the direction of load and displacement is downward; stress–strain curve; (**c**) micrographs of the fracture surfaces of archaeological wood; (**d**) SEM image of archaeological wood.

The extraction experiment of the epoxy resin multilayer material was conducted in the South China Sea, China. The archaeological wooden artifacts, which served as the objects of extraction, originated from an ancient wooden shipwreck dating back approximately 800 years and have been identified as Pinus spp. The maximum water content (MWC) of samples is about 341.11%, indicating moderate degradation. As shown in Figure 9a,b, the fracture strain of the samples is concentrated within the range of 0.73% to 0.91%. The average flexural strength is only 2.7 MPa, which is significantly lower than that of healthy modern Masson pine (>30 MPa). The decrease in mechanical strength of archaeological wood is related to changes in cellular structure. Figure 9c,d show the microscopic structure of the samples. Under the influence of underwater microbial erosion and environmental degradation, the hemicellulose in waterlogged wood degrades, causing the sample’s cell walls to thin and porosity to increase, resulting in deterioration of mechanical properties. To illustrate the protective effect of the multilayer materials on decayed artifacts and the feasibility of underwater extraction techniques, these samples were selected as the representative of underwater fragile artifacts, to carry out simulated extraction experiments.

**Figure 10:**
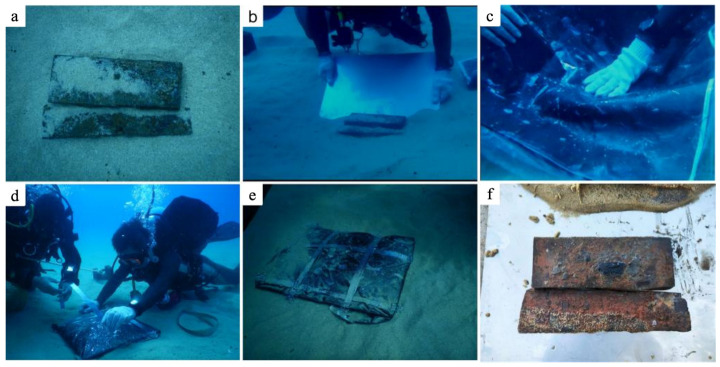
Application of the epoxy resin/carbon fiber multilayer material for the extraction of underwater archaeological wooden artifacts conducted in the South China Sea at a depth of 13 m. (**a**) the wooden artifact in its original state; (**b**) insertion of a steel plate beneath the artifact; (**c**) wrapping with the epoxy resin multilayer material; (**d**) fixation of steel strips around the artifact; (**e**) the state of the wooden artifact after encapsulation; and (**f**) the condition of the wooden artifact after being retrieved to the surface.

The experiment was conducted at a depth of 13 m, where the seawater temperature was approximately 26 °C. To ensure that the epoxy resin multilayer material remained in a highly elastic state under these conditions, the formulation was selected such that its glass transition temperature (Tg) was slightly lower than the seawater temperature. The epoxy resin/carbon fiber multilayer material prepared using the E44/PC10 formulation (Tg = 21.73 °C) was employed to extract wooden artifacts. The step-by-step process is illustrated in Figure 10: (a) the wooden artifact in its original state; (b) insertion of a steel plate beneath the artifact; (c) wrapping with the epoxy resin multilayer material; (d) fixation of steel strips around the artifact; (e) the state of the wooden artifact after encapsulation; and (f) the condition of the wooden artifact after being retrieved to the surface. After the removal of the multilayer coating, no surface damage, displacement, or morphological distortion was observed. PC addressed the issues of poor fluidity and coating performance, enhancing preparation efficiency. The homogenized multilayer materials realize the random wrapping of irregular artifacts, eliminating the occurrence of a hollow. The underwater extraction process was completed within 10 min, and meets the requirements of underwater archaeological excavation.

## 4. Conclusions

Epoxy resin multilayer materials have strong applicability in the field of underwater artifact extraction. This material is characterized by its simple preparation, immediate usability, high strength, and quick removal after extraction. To address issues such as uneven mixing and unstable curing performance caused by high viscosity, this study utilized PC diluent to reduce the viscosity of the resin, and studied the impact on curing performance. The results indicate that the PC diluent not only improves fluidity, but also participates in the construction of the main chain, extending the gel time from 22 min to 38 min without affecting the curing time. The Tg varies between 12.28 °C and 25.80 °C. The thermal expansion behavior of the epoxy resin exhibits anisotropy; compared to E44/PC0, PC reduces the CTE values in the weft direction. The multilayer materials have excellent thermal stability and adapt well to the variable temperatures of marine environments. Selecting a Tg formulation aligned with seawater temperatures effectively ensures the stability of the material. As the PC increases, the tensile strength decreases from 21.68 MPa to 7.63 MPa, with the cured product transitioning from brittle to ductile. PC can improve the toughness of multilayer materials, protecting artifacts from external damage and enhancing their fit with irregularly shaped objects. Ultimately, the formulation E44/PC10 successfully facilitated the simulated extraction of water-saturated wooden artifacts at a depth of 13 m in the South China Sea. PC provides a direct and efficient solution for preparing epoxy resin multilayer materials with superior compatibility and more stable performance, making them applicable to the complex environments of underwater archaeology. This study demonstrates the application potential of the modified epoxy resin multilayer material in the temporary reinforcement of underwater cultural relics. Future research will further evaluate the water absorption, seawater resistance, and long-term stability of the multilayer material under actual marine environmental conditions, to ensure its durability and reliability during the extraction of large-scale artifacts.

## Figures and Tables

**Table 1:** Formula of epoxy resin blends.

Number	Component A (Phr)		Component B (Phr)	
	E44	PC	JH-5553	DMP30
E44/PC0	100	0	60	3
E44/PC5	100	5	60	3
E44/PC10	100	10	60	3
E44/PC15	100	15	60	3
E44/PC20	100	20	60	3

**Table 2:** Relaxation time and gel time of different epoxy resin gels.

Number	E44/PC0	E44/PC5	E44/PC10	E44/PC15	E44/PC20
Stable viscosity (mPa·s)	7773 ± 703	5392 ± 144	3560 ± 89	1774 ± 92	1118 ± 48
Relax time (s)	48	55	60	78	139
Workable time (min)	18	20	21	24	26

**Table 3:** TGA region of cured epoxy resin products.

Numbers	First Stage		Second Stage		Third Stage		Carbon Residue /%
	T_max_ /°C	Weight Loss/%	T_max_ /°C	Weight Loss/%	T_max_ /°C	Weight Loss /%	
E44/PC0	-	-	231	16.19	371	65.76	5.71
E44/PC5	167	9.44	244	8.08	363	68.83	3.50
E44/PC10	169	9.03	244	7.85	367	70.93	2.32
E44/PC15	167	9.68	243	6.22	364	71.91	2.97
E44/PC20	166	9.15	241	4.86	351	71.20	1.90

**Table 4:** CTE of different cured epoxy resin products.

Number	Warp Direction (10^−5^/°C)		Weft Direction (10^−5^/°C)	
	Tg_under_	Tg_above_	Tg_under_	Tg_above_
E44/PC0	2.2	2.4	2.5	5.8
E44/PC5	2.2	2.7	2.4	3.5
E44/PC10	2.2	2.3	2.5	4.6
E44/PC15	2.3	2.3	2.6	4.9
E44/PC20	1.7	2.8	2.5	5.1

**Table 5:** Mechanical properties of different cured epoxy resin products.

Number	Tensile Strength (MPa)	Flexural Strength (MPa)	Elongation at Break (%)
E44/PC0	21.68 ± 2.43	108.02 ± 5.06	8.92 ± 0.83
E44/PC5	21.35 ± 1.32	134.89 ± 4.78	10.08 ± 0.61
E44/PC10	12.42 ± 1.16	114.69 ± 3.18	10.99 ± 1.23
E44/PC15	8.94 ± 1.69	77.23 ± 4.91	86.14 ± 2.30
E44/PC20	7.63 ± 0.62	61.98 ± 3.62	96.71 ± 2.41

## Data Availability

The original contributions presented in this study are included in the article. Further inquiries can be directed to the corresponding author.

## Abbreviations

The following abbreviations are used in this manuscript:

PC	Propylene carbonate
Tg	The glass transition temperature
E44	Bisphenol A type epoxy resin
DMP30	Tris (dimethylaminomenthyl) phenol
CTE	Coefficient of Thermal Expansion
MWC	The maximum water content
